# Children Eating

**Published:** 1859-05

**Authors:** 


### CHILDREN EATING.

When a child is observed to have little or no appetite for breakfast, sickness of some kind is impending. If in addition to this indifference for food in the morning, there is a uniform desire for a hearty supper at the close of the day, a dyspepsia for life will be founded, which will embitter many an otherwise happy hour; or some other form of chronic disease will result, which medical skill for many years, will often fail to eradicate.

This want of appetite in the morning, and this over appetite late in the day, is the creator of disease in multitudes of grown persons who have reached maturity in good health, but whose change of position, of business, or of associations, has gradually led to the perversion of nature's laws.

Young children naturally, in common with the animal creation, are greedy for breakfast, after the long abstinence of twelve hours, this is the natural arrangement, and it is wise.

As persons of any intelligence at all, cannot but know that eating heartily late in the day, is destructive of health, we need not stop here to prove it; but by pointing out an easy remedy, we will, if it is attended to by every reader, arrest more disease, and save more life than can easily be computed. The importance of attending to what we shall say is such, that we entreat all parents who have any true wisdom and affection, who have an abiding desire for the future happiness of their offspring, to give it their mature consideration, their steady and prompt attention.

Allow nothing to be eaten between meals, not an atom of any thing, and let the time of eating be fixed, and regular to a minute almost, for nature loves regularity.

On the first evening, allow the child just half of his common supper. In three or four days, diminish the last allowance one half more. For another week allow nothing at all, but one or two ordinary slices of cold bread and butter, and a cup of hot water and milk, with sugar in it, called cambric tea, from its similarity in color to that fabric. Meanwhile, the appetite for breakfast will gradually increase, until it becomes a hearty meal, and all the exercise of the day will go to its thorough digestion, and perfect adaptation to the nutrition of the whole system.

It is contrary to physiological law, to nature and to common sense, to eat an atom of any thing later than an hour after sun down, and alike contrary to all these is it, to make the last meal of the day, the heartiest one, as in the manner of five o'clock dinners.

Those who do not work hard, and who will have supper, "Tea," should, especially where there are children, have nothing more inviting than cold bread and butter; "Relishes," such as cake or chipped beef and the like, however "simple" they may be, are only stimulants to eating, when nature does not require any thing, and we goad her at our peril.

